# Epigenetic Suppression of the T-box Subfamily 2 (*TBX2*) in Human Non-Small Cell Lung Cancer

**DOI:** 10.3390/ijms20051159

**Published:** 2019-03-07

**Authors:** Eliana Nehme, Zahraa Rahal, Ansam Sinjab, Athar Khalil, Hassan Chami, Georges Nemer, Humam Kadara

**Affiliations:** 1 Department of Biochemistry and Molecular Genetics, Faculty of Medicine, American University of Beirut, Riad El Solh 1107 2020 Beirut, Lebanon; esn00@mail.aub.edu (E.N.); as216@aub.edu.lb (A.S.); aak67@aub.edu.lb (A.K.); gn08@aub.edu.lb (G.N.); 2 School of Medicine, American University of Beirut, Riad El Solh 1107 2020 Beirut, Lebanon; zar03@aub.edu.lb; 3 Department of Internal Medicine, Faculty of Medicine, American University of Beirut, Riad El Solh 1107 2020 Beirut, Lebanon; hchami@aub.edu.lb; 4 Department of Translational Molecular Pathology, The University of Texas MD Anderson Cancer Center, Houston, TX 77030, USA

**Keywords:** non-small cell lung cancer, *TBX2*, epigenetic suppression, methylation

## Abstract

(1) The *TBX2* subfamily of transcription factors (*TBX*s 2, 3, 4 and 5) are markedly down-regulated in human non-small cell lung cancer (NSCLC) and exert tumor suppressor effects in lung malignancy. Yet, mechanisms underlying suppressed expression of the *TBX2* subfamily in NSCLC are elusive. Here, we interrogated probable epigenetic mechanisms in suppressed expression of the *TBX2* subfamily in human NSCLC. (2) *TBX2* subfamily gene expression and methylation levels in NSCLC and normal lung tissues were surveyed using publicly available RNA-sequence and genome-wide methylation datasets. Methylation β-values of the four genes were statistically compared between NSCLCs and normal lung tissues, correlated with gene expression levels, and interrogated with clinicopathological variables. Expression and methylation levels of *TBX*s were quantified in NSCLC cells using real-time PCR and methylation-specific PCR assays, respectively. Effects of the DNA methyltransferase inhibitor 5-azacytidine (Aza) on *TBX2* subfamily expression were assessed in NSCLC cells. Impact of *TBX2* subfamily expression on Aza-treated cells was evaluated by RNA interference. (3) All four *TBX*s were significantly hypermethylated in NSCLCs relative to normal lung tissues (*p* < 0.05). Methylation β-values of the genes, with exception of *TBX2*, were significantly inversely correlated with corresponding mRNA expression levels (*p* < 0.05). We found no statistically significant differences in hypermethylation levels of the *TBX2* subfamily by clinicopathological features including stage and tobacco history. Expression levels of the *TBX* genes were overall suppressed in NSCLC cells relative to normal alveolar cells. Members of the subfamily were significantly hypermethylated in all tested NSCLC cell lines relative to normal alveolar cells. Treatment with Aza induced the expression of the *TBX2* subfamily concomitant with NSCLC cell growth inhibition. Further, simultaneous knockdown of the four *TBX* genes markedly reduced anti-growth effects of Aza in NSCLC cells. (4) Our study sheds light on new epigenetic profiles in the molecular pathogenesis of human NSCLC.

## 1. Introduction

Lung cancer is the leading cause of cancer-related deaths in both men and women worldwide and accounts for more deaths than breast, colorectal, and prostate cancers combined [1]. While significant advances have been made in lung cancer therapy, the overall 5-year survival rates of this malignancy remain very poor, not exceeding 15% across all stages [2]. These low survival rates are largely due to late diagnosis at advanced stages in which tumors have already metastasized [2]. Lung cancer is broadly classified into two major histological types based on morphological criteria: non-small cell lung cancer (NSCLC) and small cell lung cancer (SCLC) [3]. NSCLC, the most common type of lung cancer, accounts for approximately 85% of all lung malignancies and is histologically subdivided into adenocarcinoma, squamous cell carcinoma, and large cell carcinoma [3]. 

The T-box family is a group of evolutionarily conserved transcription factors playing vital roles in development, differentiation, and organogenesis [4], particularly in the respiratory system [5,6,7,8]. The *TBX2* subfamily members (*TBX*2, 3, 4 and 5) are markedly expressed in murine developing lung buds and trachea [6]. Also, genetic deletion of members of the *TBX2* subfamily in the mouse lung inhibits proper branching morphogenesis [7]. The study by Arora and colleagues demonstrated that the *TBX2* subfamily members *TBX4* and *TBX5* are crucial for bronchial differentiation [5]. Despite the significant functions the *TBX2* subfamily members play in murine lung development and organogenesis, their roles in malignancies of the lung are otherwise very poorly understood. A recent study by Khalil and colleagues showed that all four members of the *TBX2* subfamily are not only preferentially expressed in normal lung compared to normal tissues from other organs but are also markedly, and commonly, suppressed in both human premalignant and malignant lung lesions compared to uninvolved normal lung tissues [9]. In another study, over-expression of each of the four members of the *TBX2* subfamily independently inhibited cell growth and proliferation as well as induced apoptosis in NSCLC cell lines [10]. 

Despite recent reports implicating members of the *TBX2* subfamily as potential tumor suppressors in the lung largely by virtue of their reduced expression, the mechanisms by which these genes are suppressed in human NSCLC are still poorly understood. Here, we interrogated epigenetic silencing, namely hypermethylation, as a high-potential mechanism underlying suppressed expression of the *TBX2* subfamily in human NSCLC.

## 2. Results

### 2.1. Hypermethylation and Suppressed mRNA Expression of the TBX2 Subfamily in Human NSCLC

Recent studies have shown that mRNA levels of the four members of the *TBX2* subfamily are markedly decreased in both preneoplastic and neoplastic lesions (NSCLCs) in the human lung suggestive of tumor suppressor properties for these genes [9]. Here we sought to examine the role of epigenetic mediated suppression by hypermethylation of the four members of the *TBX2* subfamily in human NSCLC. 

**Figure 1:**
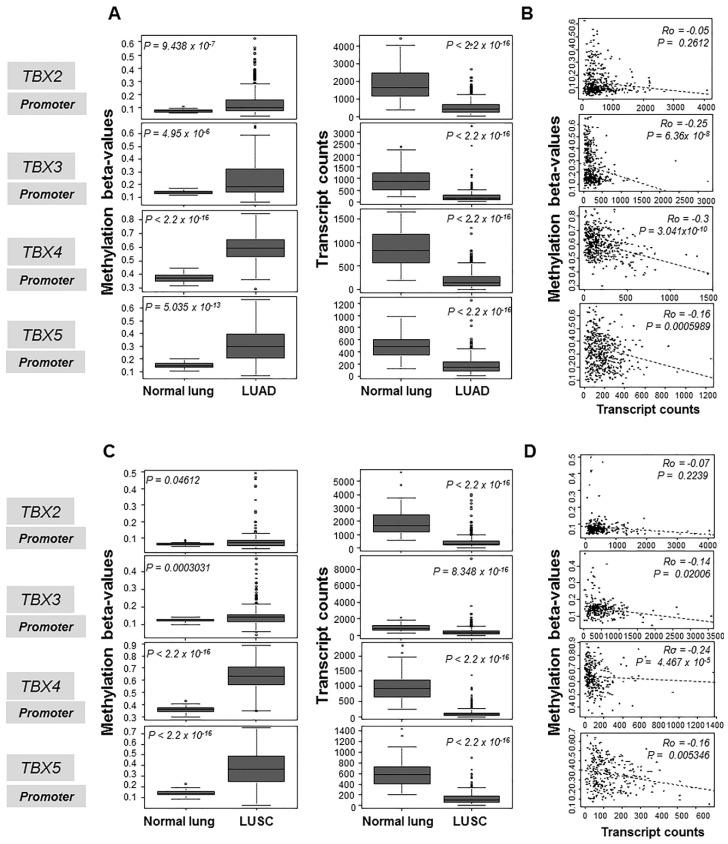
Increased promoter-level methylation of the *TBX2* subfamily in human LUADs and LUSCs compared to normal lung tissues. Promoter-level methylation β-values (left panels) and mRNA levels (right panels) in 460 LUADs (**A**,**B**) and 370 LUSCs (**C**,**D**) were propagated from the MethHC database of methylation and gene expression in cancer. Methylation β-values and mRNA levels of the *TBX* genes were statistically analyzed by the Wilcoxon rank sum test in LUADs relative to normal lung tissues (**A**). Methylation β-values and mRNA levels of the *TBX* genes were also similarly statistically analyzed LUSCs relative to normal lung tissues (**C**). Methylation β-values and mRNA expression levels for each of the four *TBX* genes were statistically correlated in LUADs (**B**) and in LUSCs (**D**) using Spearman’s correlation. Statistical analyses and plots were performed and generated using the R language and environment.

We first interrogated available data of human NSCLC gene expression (Illumina RNA-sequencing) and methylation β-values (Illumina Infinium methylation arrays) consisting of 460 LUADs and 370 LUSCs from the MethHC database of methylation and gene expression in cancer [11]. We propagated data from multiple promoter and CpG island probes for each of the four *TBX* genes and statistically examined differences in methylation β-values for each gene among NSCLCs and normal lung tissues, separately for LUADs and LUSCs, and in association with mRNA expression levels. Analysis of a specific promoter probe in the LUAD cohort revealed that the four *TBX* genes displayed markedly increased methylation β-values in the tumors compared to normal lung tissues indicative of hypermethylation (*p* < 10^−5^; Figure 1A, left panels). Conversely, mRNA expression levels of each of the four *TBX* genes were significantly down-regulated in LUADs relative to normal lung tissues (*p* < 10^−15^; Figure 1A, right panels). Methylation β-values were overall (with exception of *TBX2*) significantly inversely correlated with corresponding mRNA expression levels (*p* < 10^−3^, Figure 1B). Similar results were obtained in LUADs when propagating methylation β-values from CpG islands (Figure S1). We next analyzed methylation and expression levels of the *TBX* genes in human LUSCs. At the promoter level, all four *TBX* genes displayed significantly elevated methylation β-values when compared to normal lung tissues (*p* < 0.05; Figure 1C, left panels). Similar to LUADs, mRNA expression levels exhibited opposing patterns with levels significantly decreased in the tumors compared to normal lung tissues (*p* < 10^−15^; Figure 1C, right panels). Methylation β-values for the genes, with exception of *TBX2,* were significantly inversely correlated with corresponding mRNA expression levels (*p* < 0.05, Figure 1D). Similar results were obtained in LUSCs when propagating methylation β-values from CpG islands (Figure S2). It is noteworthy to mention that *TBX4* and *TBX5* displayed elevated methylation β-values compared with *TBX2* and *TBX3* in both LUADs and LUSCs as well as more pronounced mRNA suppression (Figure 1, Figures S1 and S2). These data suggest that the four members of the *TBX2* subfamily, particularly *TBX4* and *TBX5*, are hypermethylated, while having reduced mRNA expression levels, in clinical human NSCLC samples. These findings point to aberrant epigenetic silencing as a potential mechanism for suppressed expression of the *TBX* genes in human NSCLC. 

### 2.2. Association of TBX2 Subfamily Hypermethylation with Clinicopathological Features in Human Clinical NSCLC Samples

**Figure 2:**
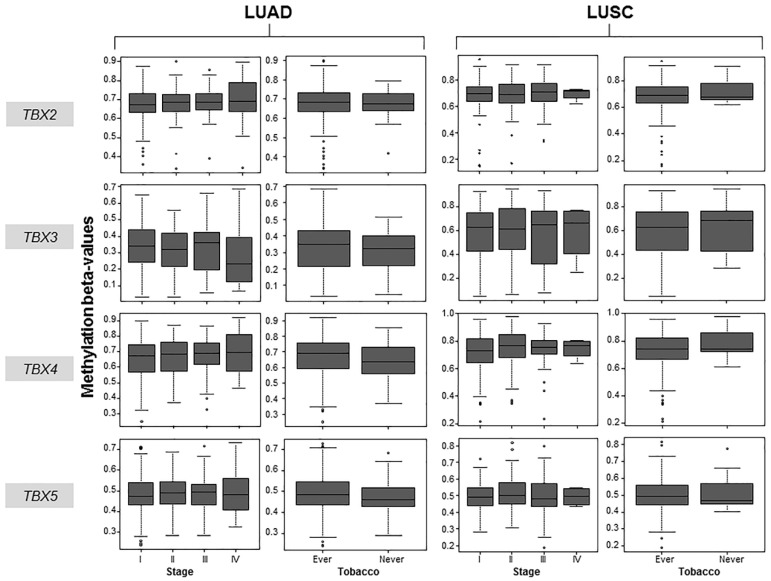
Association of *TBX2* subfamily hypermethylation with clinicopathological features in human LUAD and LUSC. Gene-level methylation β-values in 522 LUADs (two left panels) and 504 LUSCs (two right panels) were propagated from the TCGA and statistically interrogated with pathological stage and tobacco history (ever: lifetime smokers, never: non-smokers). Kruskal-Wallis test was used to statistically examine differences in methylation β-values of the *TBX* genes by pathological stage. Wilcoxon rank sum test was used to statistically analyze methylation β-values based on tobacco exposure. All statistical analyses and plots were performed and generated using the R language and environment.

We next sought to clinically contextualize hypermethylation of each of the four *TBX* genes in the human NSCLC samples. *TBX2* subfamily gene expression counts and gene-level methylation β-values in clinically annotated LUADs and LUSCs were obtained from the TCGA (*n* = 1,026 NSCLCs; 522 LUADs and 504 LUSCs). We statistically analyzed methylation β-values of each of the four *TBX* genes, separately for LUADs and LUSCs, in association with pathological stage (stages I, II, III, IV), gender (female, male), tobacco history (lifetime smoker, non-smoker), age and ethnicity (Asian, African American, White). We found no statistically significant differences among methylation β-values for each of the four genes in LUADs and LUSCs based on pathological stage (Figure 2). These data suggest that hypermethylation of the *TBX2* subfamily may be an early event since it is equally prevalent among early- and advanced-stage NSCLCs. Similarly, there were no statistically significant differences in *TBX2* subfamily methylation among NSCLCs by smoking, gender, age nor ethnicity, perhaps suggesting that hypermethylation of these transcription factors may be a prevalent and common feature in NSCLCs (Figure 2 and Figure S3). These data raise the possible suggestion that hypermethylation of the *TBX2* subfamily occurs early in the pathogenesis of NSCLC and is a common molecular event, and thus a potential marker, in this malignancy. 

### 2.3. Hypermethylation and Suppressed mRNA Expression of TBX2 Subfamily Members in NSCLC Cell Lines Relative to Normal Alveolar Cells (NAC)

**Figure 3:**
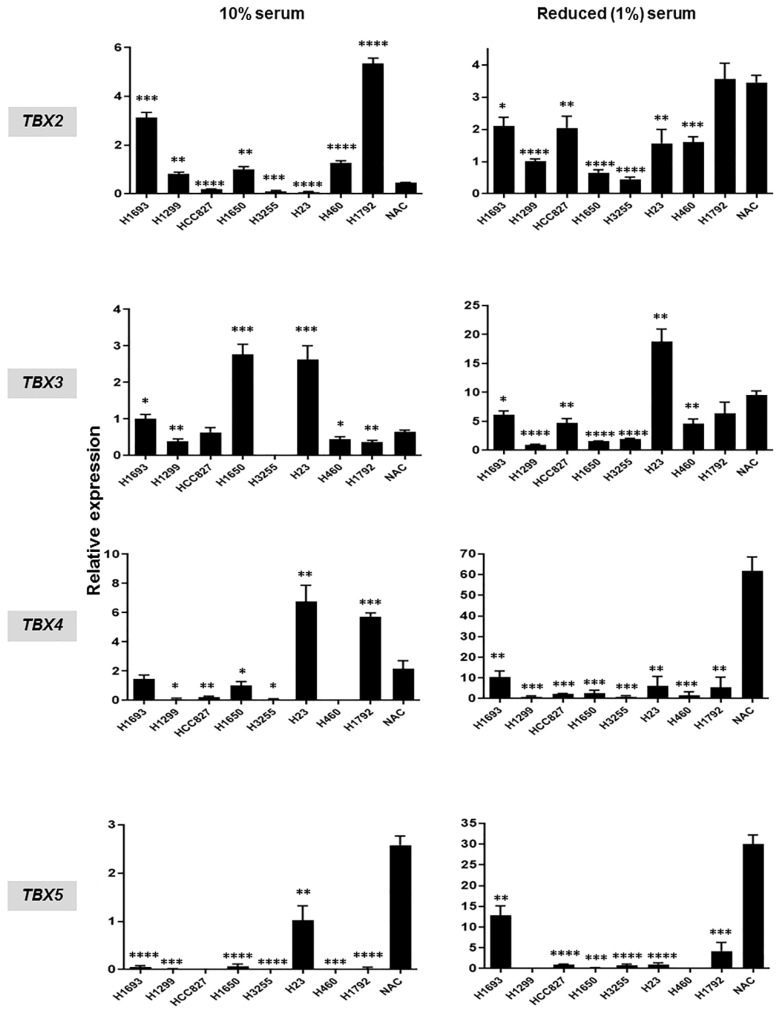
Suppressed expression of *TBX2* subfamily in non-small cell lung cancer (NSCLC) cell lines relative to normal pulmonary alveolar epithelial cells. Messenger RNA expression levels of all four members of *TBX2* subfamily in NSCLC cell lines compared to normal pulmonary alveolar epithelial cells (NAC) were analyzed in triplicates by qRT-PCR. Analysis was performed on NSCLC cell lines grown in serum-containing medium (10% FBS; left panels) as well as on cells maintained in media with reduced serum (1% FBS; right panels). Relative expression data in each of the NSCLC cell lines and for each of the *TBX* genes were computed using the 2^−ΔΔ*C*t^ calculation method by normalization to glyceraldehyde 3-phosphate dehydrogenase *(GAPDH)* and are presented as mean ± SEM (* *p* < 0.05; ** *p* < 0.01; *** *p* < 0.001; **** *p* < 0.0001) compared to normal alveolar epithelial cells (NAC).

Following our in silico analysis, we sought to concomitantly examine mRNA expression and DNA methylation levels of the four *TBX* genes in human NSCLC cell lines. We first compared and contrasted mRNA expression levels, by quantitative real-time PCR (qRT-PCR), in eight NSCLC cell lines, relative to normal alveolar epithelial cells (NAC). Analysis was first performed using NSCLC lines cultured in serum-containing medium (10% fetal bovine serum: FBS). We found that mRNA levels of the four *TBX* genes were, overall, decreased in the NSCLC cell lines relative to NAC (Figure 3; left panels). When compared to NAC, not all NSCLC cell lines displayed reduced mRNA levels of the *TBX* genes (Figure 3; left panels). To achieve a more suitable comparison of gene expression between NSCLC cell lines and NAC, we analyzed RNAs from the same eight NSCLC cell lines but that were instead cultured in reduced serum (1% FBS) medium, since NAC are typically grown in reduced serum conditions. This analysis demonstrated that NSCLC cell lines cultured in reduced serum also exhibited, overall, reduced *TBX* mRNAs compared to NAC (Figure 3; right panels). In accordance with our in silico analysis of clinical NSCLCs, mRNA levels of *TBX4* and *TBX5* exhibited more pronounced suppression in NSCLC cell lines, particularly when cells were cultured in reduced serum conditions, compared with levels of *TBX2* and *TBX3* (Figure 3).

**Figure 4:**
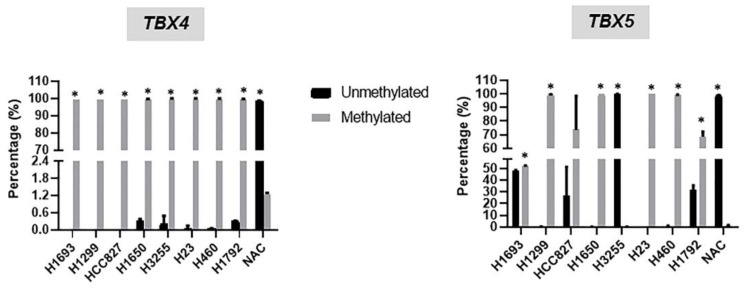
Members of the *TBX2* subfamily are hypermethylated in NSCLC cell lines. Methylation levels of members of the *TBX2* subfamily were examined in eight NSCLC cell lines and normal alveolar epithelial cells by quantitative methylation-specific PCR using the Epitect Methyl II PCR assay from QIAGEN according to the manufacturer’s instructions (see Section 4). Percentages of un-methylated (UM) and methylated (M) *TBX2* subfamily alleles are depicted and represent an average of two experiments (* *p* < 0.05 comparing methylated versus unmethylated fractions for each cell line).

We then probed the extent of epigenetic suppression by hypermethylation of the *TBX* genes in the NSCLC cell lines. To achieve this, we performed methylation-specific polymerase chain reaction assays (MSPs) of the NSCLC cell lines relative to NAC to quantitatively assess unmethylated and methylated allelic fractions of *TBX4* and *TBX5,* which we found above to be the most methylated members of the *TBX2* subfamily in NSCLC. The MSP analysis demonstrated that the studied NSCLC cell lines with the exception of H3255, exhibited, overall, significant hypermethylation of both *TBX4* and *TBX5* relative to NAC (Figure 4). The average (of duplicates) of unmethylated and methylated fractions (shown as percentages) of *TBX4* and *TBX5* are shown in Table S1. The methylated levels of the genes in the NSCLC cell lines excluding the H3255 line ranged from 46.5% to 100% and the unmethylated fractions in the same tumor cells ranged from 0% to 53.5% (Figure 4). In sharp contrast, there was very minimal, if any, methylation of the *TBX* genes in NAC (methylation fraction: 1.9%; unmethylated: 98.1%). These data suggest that, very synonymous to the clinical data above, hypermethylation of members of the *TBX2* subfamily commonly occurs in NSCLC cell lines and is absent in normal alveolar epithelial cells.

### 2.4. Induction of TBX2 Subfamily Expression following Treatment with the DNA Methyltransferase Inhibitor 5-Azacytidine

**Figure 5:**
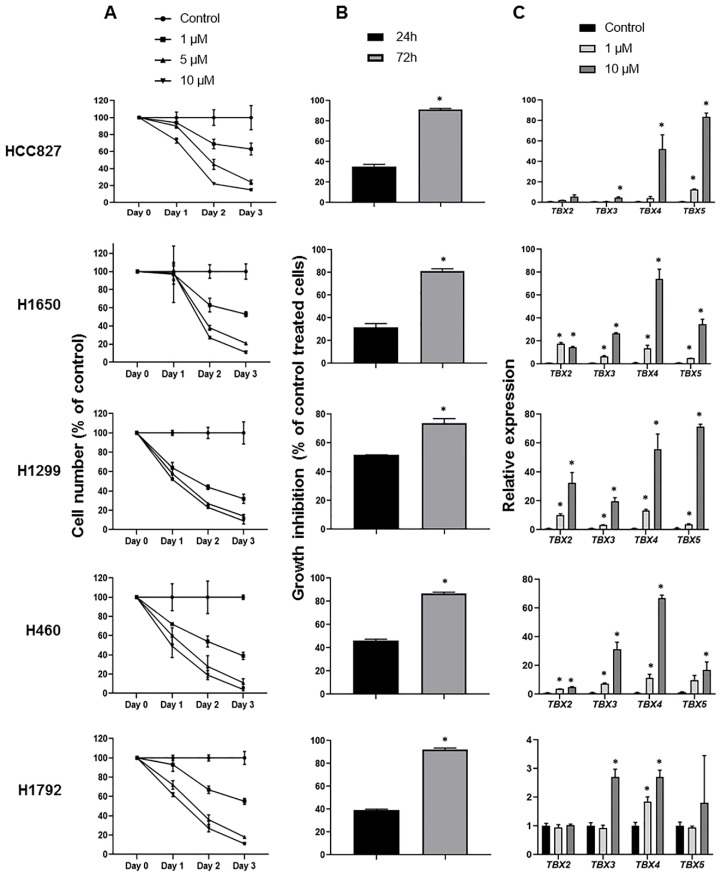
Augmented expression of the *TBX2* subfamily in NSCLC cell lines treated with the DNA methyltransferase 5-azacytidine. (**A**) 3-(4,5-dimethylthiazol-2-yl)-2,5-diphenyltetrazolium bromide (MTT) assays were performed to assess cellular proliferation as described in Section 4. Cells were seeded in 96-well plates, at varying seeding densities following optimization, and treated with differing doses of Aza or dimethyl sulfoxide (DMSO) as control for 24, 48 and 72 h. Aza treatment was prepared and replenished every day as per previous reports [14]. Cell numbers (% of control) were determined by MTT assays and represent percentages of absorbance readings from Aza-treated cells (at each dose) versus DMSO-treated cells. Data depicted represent means ± standard deviations. (**B**) The same NSCLC cell lines were seeded in 96-well plates and treated according to IC50s (1 µM) for 24 h and 72 h. Assessment of growth inhibition was performed using the trypan blue dye exclusion method as detailed in Section 4. Growth inhibition was calculated according to the following formula: 100 − (treated/non-treated) × 100). Data depicted comprise the means ± SEM of two independent experiments (* *p* < 0.05). (**C**) Messenger RNA expression levels of all four members of *TBX2* subfamily in control and Aza-treated NSCLC cell lines (triplicates) were analyzed by qRT-PCR using the 2^−ΔΔ*C*t^ calculation method by normalization to *GAPDH* and are presented as mean ± SEM (* *p* < 0.05).

We next aimed to determine whether the hypermethylated state of the *TBX2* subfamily in the NSCLC cell lines may be reversed, or at least attenuated, by inhibition of DNA methyltransferases. To do so, we evaluated the expression of *TBX* genes in NSCLC cells treated with the pharmacological epigenetic modulator 5-azacytidine (Aza) in comparison to control-treated cells. In association with *TBX* gene expression analysis, we also concomitantly examined the anti-proliferative and -growth effects of Aza on the NSCLC cells, since inhibitory effects of Aza have been previously reported in lung tumor models [12,13]. We performed MTT assays (see Section 4) to examine the effect of Aza, in comparison to control DMSO-treated cells, on the proliferation rates of five NSCLC cell lines. In line with previous reports [12,13], Aza treatment resulted in significant (all *p* < 0.05) dose- and time-dependent decreases in cell proliferation of all NSCLC cell lines examined (Figure 5A). Similarly, trypan blue dye exclusion assays demonstrated that Aza, at both 24 h and 72 h post-treatment, significantly (all *p* < 0.05) reduced cell growth across all five NSCLCs examined (Figure 5B). Concomitant with these anti-proliferative and -growth effects, Aza treatment resulted, overall, in markedly increased/induced expression of the *TBX2* subfamily genes at 72 h post-treatment and in a dose-dependent fashion (Figure 5C). Induction of the *TBX* genes by Aza was evident in all NSCLC cell lines tested (Figure 5C). Also in line with our above analyses, induction of *TBX4* and *TBX5* mRNA by Aza treatment in NSCLC lines was more pronounced relative to *TBX2* and *TBX3*. These results lend further evidence to hypermethylation of the *TBX2* subfamily, particularly *TBX4* and *TBX5*, in NSCLC cells. These findings demonstrate that treatment with the DNA methyltransferase inhibitor Aza increased the expression of all four members of the *TBX2* subfamily, particularly *TBX4* and *TBX5*, suggestive of demethylation of the genes by the drug.

### 2.5. Impact of TBX2 Subfamily Knockdown on Anti-Growth Effects of 5-Azacytidine in NSCLC Cells

**Figure 6:**
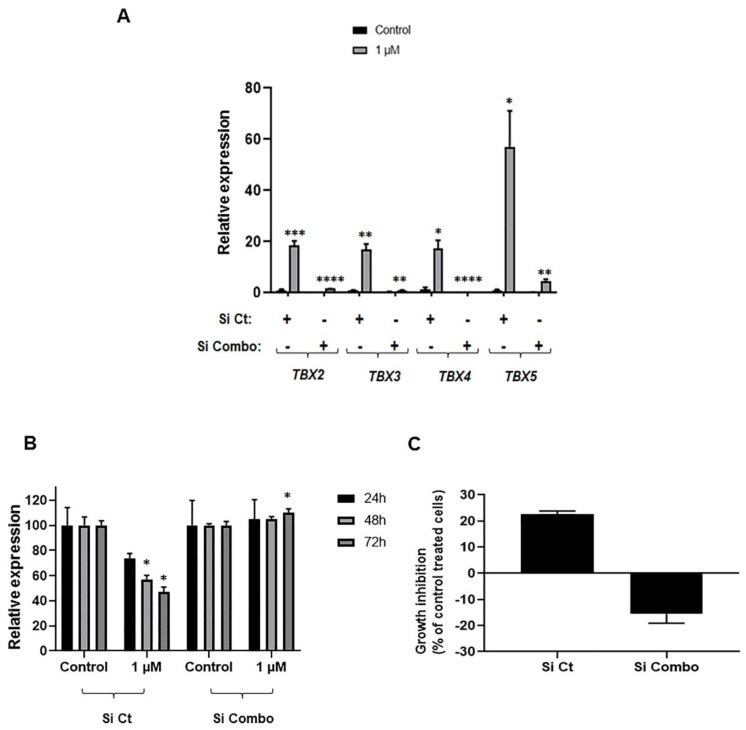
Impact of simultaneous knockdown of all four members of *TBX2* subfamily on anti-growth effects of Aza in H1299 cells. (**A**) H1299 cells were transfected with control (4× scrambled) siRNAs and siRNAs targeting all four members of the *TBX2* subfamily simultaneously (combination of four target-specific siRNAs) using Lipofectamine RNAiMAX as described in Section 4. The day following transfection, cells were re-seeded in six cm dishes. The next day cells were incubated overnight with reduced serum (1% FBS) medium prior to treatment with either DMSO control or 1 µM Aza for 72 h. Total RNA was extracted and RNA expression levels of *TBX2, TBX3, TBX4* and *TBX5* were analyzed using the 2^−∆∆*C*t^ calculation method by normalization to *GAPDH*. H1299 cells were transfected in the same manner described above, except they were re-seeded the following day in 96-well plates for MTT assays (**B**) and trypan blue exclusion analysis (**C**). Cell numbers (% of control DMSO treatment) were determined using MTT assays (**B**) and percentages of cell growth inhibition in Aza-treated cells were obtained using the trypan blue exclusion method (**C**). Growth inhibition following the trypan blue exclusion analysis was calculated using the following formula: (100 − (treated/non-treated) × 100) and is depicted for the Aza-treated cells. Data depicted represent mean ± SD for MTT assays and mean ± SEM of triplicates for trypan blue exclusion analysis (* *p* < 0.05).

We next sought to begin to probe the functional relevance of induced *TBX2* subfamily expression to the anti-growth effects of Aza in NSCLC cells. To achieve this, we first compared and contrasted the anti-growth effects of Aza in H1299 cells transfected with scrambled small interfering RNAs (siRNAs) to cells transfected with siRNAs individually targeting *TBX2* subfamily members, namely *TBX4* and *TBX5*. Analysis was also conducted in comparison to control DMSO-treated cells transfected with scrambled and target-specific siRNAs. While target-specific siRNAs effectively suppressed expression of *TBX4* and *TBX5* in Aza treated cells (Figure S4), there were no significant differences in proliferation and growth rates between Aza (or DMSO)-treated cells (1 µM Aza) transfected with scrambled siRNAs and treated cells with siRNAs individually targeting *TBX4* and *TBX5* (Figure S5). Drawing from our observations that all four members of the *TBX2* subfamily were hypermethylated collectively in NSCLC cells, we surmised that individual knockdown of the *TBX* genes may not be sufficient to rescue cells from anti-growth effects of Aza. Indeed, we found by further qRT-PCR analysis that the other three *TBX2* subfamily members following *TBX4* or *TBX5* knockdown were still markedly induced by Aza treatment (Figure S6). Given these observations, we next sought to assess the effects of simultaneous siRNA-mediated knockdown of all four members of the *TBX2* subfamily on control- and Aza-treated cells. Combinatorial and simultaneous siRNA-mediated knockdown of the *TBX2* subfamily (denoted by “Si Combo”) efficaciously abrogated Aza-mediated induction of all four genes when compared with cells transfected with control/scrambled siRNAs (Figure 6A). Of note, combinatorial knockdown of all four members of the *TBX2* subfamily markedly reduced the anti-proliferative (Figure 6B) and anti-growth (Figure 6C) effects of 1 µM Aza. These findings demonstrate that the *TBX2* subfamily is collectively important for the anti-growth effects of the DNA methyltransferase inhibitor and cancer treatment agent 5-azacytidine in NSCLC cells. 

## 3. Discussion

In this study, we interrogated the role of epigenetic silencing in suppressed expression of the *TBX2* subfamily of transcription factors in NSCLC. By in silico analysis of a cohort of NSCLCs and normal lung tissues, we first found that all four members of the *TBX2* subfamily were, overall, hypermethylated, at both promoter regions and CpG islands, in clinical NSCLC samples compared to normal lung. Methylation β-values for the genes, with exception of *TBX2*, were overall significantly inversely correlated with corresponding mRNA expression levels. Also, we found no statistically significant differences in hypermethylation levels of the *TBX2* subfamily between early- and advanced-stage tumors. In our in vitro studies, we found that NSCLC cell lines commonly displayed significant hypermethylation concomitant with suppressed gene expression of the *TBX2* subfamily members relative to normal alveolar epithelial cells. Further, treatment with the DNA methyltransferase inhibitor Aza inhibited proliferation and growth of all tested NSCLC cell lines while concomitantly significantly augmenting the expression of all four members of the *TBX2* subfamily. Lastly, we showed that simultaneous RNA-interference mediated knockdown of all four members of the *TBX2* subfamily members markedly attenuated the anti-growth effects of Aza. Our study provides new insights on therapeutically pliable and aberrant epigenetic mechanisms in the molecular pathogenesis of NSCLC involving prevalent hypermethylation of the *TBX2* subfamily of evolutionarily conserved transcription factors. 

Our in silico analysis demonstrated significant hypermethylation of the four members of the *TBX2* subfamily, particularly *TBX4* and *TBX5*, in both LUAD and LUSC relative to normal lung tissues. When analyzing methylation β-values of the genes in the context of clinicopathological attributes we found no significant differences in the methylation values by stage; meaning hypermethylation of the genes was similar between NSCLCs of early (stages I and II) and relatively more advanced (stages III and IV) stages. It is thus plausible that hypermethylation of the *TBX2* subfamily occurs early on in the progression of NSCLC. Our analysis also showed that *TBX2* subfamily hypermethylation was similar between lifetime smokers and non-smokers, by gender as well as by clinicodemographic variables like ethnicity. Given our findings, it is reasonable to assume that hypermethylation of these genes is a common molecular feature of NSCLCs and indirectly lends testament to the notion that this hypermethylation occurs early on in the linear/parallel evolution of lung malignancy. It is not clear whether the epigenetic suppression of *TBX*s is conserved and found in mouse tumors, given that *TBX2* subfamily members play crucial roles in development and organogenesis in mice [15]. Further, the DNA methylation patterns of these genes based on NSCLC driver mutated gene status are not well understood. Future studies are warranted to fill these voids and to determine, for instance, whether *TBX2* subfamily methylation levels differ by mutational status of key driver genes—though it is noteworthy to highlight what we have shown here, that methylation of these genes is a common event in NSCLC cell lines with likely disparate mutated drivers. Nonetheless, given the pragmatic application of DNA methylation profiles as non-invasive biomarkers [16], our observations on the prevalence of *TBX2* subfamily hypermethylation in NSCLC suggest that methylation of these genes may serve as a high-potential biomarker for early detection and/or diagnosis of lung cancer. 

Using methylation-specific PCR analysis we showed that different members of the *TBX2* subfamily were, overall, hypermethylated in all NSCLC cell lines examined relative to normal alveolar cells. A number of studies evaluated the significance of promoter methylation of tumor suppressor genes in the development of lung cancer. Our study showing hypermethylation of members of the *TBX2* subfamily is in line with previous studies underscoring specifically *TBX5* methylation in lung tumors [17] and sheds more light on the epigenome of NSCLC. It is not clear whether hypermethylation of the *TBX2* subfamily is specific to NSCLC or may be common to several tumor types. Several studies support the latter argument. For instance, methylation of *TBX2* and *TBX3* were shown to be associated with prognosis and predict progression in bladder cancer [18,19]. *TBX5* was also shown to be epigenetically inactivated by promoter methylation in colon cancer [20]. It is important to mention that other mechanisms may account for epigenetic suppression of the *TBX2* subfamily and underlie their reduced RNA expression levels (e.g., histone modifications). Future studies are warranted to probe other mechanisms governing epigenetic inactivation of the *TBX2* subfamily.

We found that treatment of NSCLC cell lines with the DNA methyltransferase inhibitor Aza inhibited the proliferation and growth of NSCLC cell lines tested, in line with previous reports [12,13], concomitantly with augmented expression of the *TBX* genes in a dose- and time-dependent manner. These observations lend further support to the supposition that the *TBX2* subfamily is epigenetically suppressed by hypermethylation in NSCLC. In our efforts to understand mechanisms governing suppression of the *TBX2* subfamily in NSCLC, we herein provide new insights into the mechanisms of action of the anti-tumor drug Aza in this malignancy. We found that simultaneous knockdown by RNA interference of all four members of the *TBX2* subfamily markedly decreased anti-growth effects of Aza treatment when compared with cells treated with the agent but transfected with scrambled siRNAs. In line with our observations albeit indirectly, a recent study by Khalil and colleagues showed that over-expression of individual members of the *TBX2* subfamily resulted in gene signatures that embody global demethylation suggesting that the genes themselves influence downstream epigenetic mechanisms [10]. Given our findings on the importance of *TBX2* subfamily induction to the anti-growth effects of Aza, it is plausible that these *TBX* genes may play crucial roles in the epigenomic circuitry of NSCLC. It is also conceivable that methylation status of the *TBX2* subfamily may serve as a predictive marker for response of NSCLCs or other cancer types to the anti-tumor drug Aza. 

It is interesting to note that of the four *TBX*s, *TBX4* and *TBX5*, despite not being linked on the same chromosomal region, exhibited overall more pronounced mRNA suppression in the NSCLC cell lines compared to *TBX*s 2 and 3. Similarly, suppression of *TBX*s 4 and 5 was more pronounced in clinical NSCLC samples, relative to normal lung, when compared with *TBX*s 2 and 3. In accordance, treatment with Aza resulted in, overall, higher *TBX4* and *TBX5* mRNA levels relative to *TBX*s 2 and 3. While these patterns are interesting, the mechanisms underlying them are not clear and are yet to be determined. It is worthwhile to mention that the study by Arora and colleagues in the mouse revealed that *Tbx4* and *Tbx5* play crucial roles in bronchial differentiation and lung branching [5]. It is noteworthy that Du et al. demonstrated that *TBX5* mRNA down-regulation is a prevalent molecular feature in LUAD [21]. Further analysis by the same group showed that *TBX5* resided in significant functional integrated regulatory gene-gene interaction networks in the LUAD phenotype [21]. Moreover, Zhang and colleagues showed that *TBX5* mRNA was down-regulated across different subtypes of lung cancer including small-cell lung carcinomas [22]. Further, a genome-wide DNA methylation profiling study revealed that *TBX5* methylation is a putative marker for LUSC [17]. Also, a recent study demonstrated down-regulation of *TBX4* in carcinoma-associated fibroblasts of NSCLC [23]. Further studies are warranted to fully scrutinize the impact of *TBX4* and *TBX5* epigenetic inactivation in early phases of NSCLC.

Previous reports have shown contradictory roles for members of the *TBX2* subfamily in cancer. For instance, over-expression of *TBX2* and *TBX3* were reported in breast, bladder, liver and ovarian cancers [24,25]. In sharp contrast, recent reports by Khalil and colleagues demonstrated that members of the *TBX2* subfamily are collectively suppressed in NSCLC [9,10]. Also, various independent studies demonstrated tumor suppressor properties for specific members of the subfamily in NSCLC [10,22,26]. It is important to mention that the report by Khalil et al. demonstrated that the *TBX2* subfamily is markedly preferentially expressed in normal lung compared to other organ-specific normal tissues [9]. These observations suggest lung lineage-specific properties for the *TBX2* subfamily and may, at least in part, help explain the contrasting expression and functional patterns of these genes across different cancer types. Here, we suggest that DNA methylation may govern suppressed gene expression of the *TBX2* subfamily in tumors arising in the lung and that epigenomic mechanisms such as DNA methylation may drive the disparate patterns of expression of these genes across different cancer types. Notwithstanding, collective suppression of all four members of the *TBX2* subfamily was only reported in NSCLC [9], thus it is also conceivable that DNA methylation underlies this phenomenon. Supporting this argument is our observation that simultaneous knockdown of all four members of the *TBX2* subfamily, but not of individual *TBX*s, attenuated the anti-growth effects of the DNA methyltransferase and cancer drug 5-azacytidine.

It is important to note that our study is not without limitations. In our study, we have centered our attention on probing for gene expression and DNA methylation levels of the *TBX2* subfamily. It is not clear whether protein levels of the *TBX2* subfamily are also augmented by treatment with Aza. Future proteomic studies are warranted to determine the effects of DNA methyltransferase inhibitors on *TBX2* subfamily protein expression in NSCLC. In addition, while the induction of members of the *TBX2* subfamily by Aza was replicated across different NSCLC cell lines, we had noted variability in the extent of induction of the genes by Aza within the same cell line across different experiments. This variability is likely attributable to the biodegradable nature of Aza [14,27,28]. It is also noteworthy, that our analyses and findings do not preclude potential cross-talk, and/or antagonism between the four members of the *TBX2* subfamily. Indeed, the four members are present in both mouse and human genomes as cognate (*TBX*s 2 and 3; *TBX*s 4 and 5) and linked (*TBX*s 2 and 4, *TBX*s 3 and 5) gene pairs [6]. Thus, given the biology of these genes, it cannot be neglected that individual knockdown of one *TBX* gene, for instance in Aza-treated cells, could thus affect levels of expression of its linked and/or its cognate gene. This supposition may explain the effect of siRNA-mediated knockdown of either *TBX4* or *TBX5* in Aza-treated cells on the expression of the other three members (Figure S6). In this context, it is also plausible that individual members of the *TBX2* subfamily may be impacted disparately by Aza and, thus, exert different roles during global hypo-methylation by this DNA methyltransferase inhibitor. Future studies are warranted to fully explore the functional roles of different members of the *TBX2* subfamily, and their interplay, in lung cancer cells treated with DNA methyltransferase inhibitors. 

## 4. Materials and Methods

### 4.1. In Silico Analysis of TBX2 Subfamily Methylation and Expression in Clinical Human NSCLC Samples

*TBX2* subfamily gene expression and methylation levels in NSCLCs and normal lung tissues were surveyed using publicly available RNA-sequence and genome-wide methylation datasets that include normal tissues (*n* = 830 NSCLCs; 460 LUADs and 370 LUSCs) obtained from the MethHC database of DNA methylation and gene expression in human cancer [11]. Analysis was performed separately for LUADs and LUSCs. Methylation β-values (for each promoter or CpG island probe), as well as gene expression levels, independently for each of the four *TBX* genes were plotted and statistically compared between NSCLCs and normal lung tissues using the Wilcoxon rank sum test. Methylation β-values were statistically correlated with gene expression levels of each of the four genes using Spearman’s rank correlation. *TBX2* subfamily gene expression counts and gene-level methylation β-values in clinically annotated LUADs and LUSCs were obtained from The Cancer Genome Atlas (TCGA; *n* = 1,026 NSCLCs; 522 LUADs, and 504 LUSCs). Methylation β-values for each of the four genes and separately for LUADs and LUSCs were statistically correlated with various clinicopathological variables. The Wilcoxon rank sum test was used for analysis of clinicopathological features with two categories (e.g., gender) and the Kruskal-Wallis test was used for three or more categories. Correlation of patient age with methylation β-values was statistically interrogated using Spearman’s rank correlation. All analyses and graphical generation were performed in the R language and environment. 

### 4.2. Cell Culture

Human NSCLC cell lines used in this study included: H1299, H1693, H1792, H23, H460, H1650, HCC827 and H3255, originally obtained from the ATCC. NSCLC cell lines were cultured and maintained in Dulbecco’s Modified Eagle Media (DMEM-F12) supplemented with 10% Fetal Bovine Serum (FBS) (Sigma-Aldrich, Buchs, Switzerland) and 1% penicillin/streptomycin antibiotics (Lonza, Basel, Switzerland Cat. #DE17-602E) as well as 5 µg/mL Plasmocin™ Prophylactic (InvivoGen, SanDiego, CA, USA, Cat. #ant-mpp). In addition, normal human pulmonary alveolar epithelial cells (NAC; ScienCell, Carslbad, CA, USA, Cat. #3200), growing in alveolar epithelial cell medium (ScienCell, Carslbad, CA, USA, Cat. #3201), were used. All cell lines were cultured and maintained in a humidified atmosphere at 37 °C with 5% CO_2_. 

### 4.3. DNA and RNA Isolation

DNA was extracted from NSCLC cell lines using the QIAmp DNA mini kit (QIAGEN, Venlo, Netherlands, Cat. #51304) according to the manufacturer’s instructions. Total RNA was extracted using RNeasy plus mini kit (QIAGEN, Cat. #74136) according to the manufacturer’s instructions. DNA and RNA quantities were evaluated using the DeNovix DS-11FX Spectrophotometer according to the manufacturer’s protocol. 

### 4.4. Two-Step Quantitative Real-Time Polymerase Chain Reaction (qRT-PCR)

RNA samples were reverse-transcribed using QuantiTect Reverse Transcription Kit (QIAGEN, Cat. #205311) according to the manufacturer’s instructions. Primer sequences for the four *TBX* genes and for the housekeeping gene glyceraldehyde 3-phosphate dehydrogenase (*GAPDH*) are summarized in Table S2. cDNA was amplified in a 20 µL mixture loaded in 96-well plates containing forward and reverse primers, iTaq™ Universal SYBR^®^ Green Supermix (Bio-Rad, Hercules, CA, USA, Cat. #172-5124) and RNase-free water. The thermal cycling conditions consisted of an initial denaturation step at 95 °C for 5 min, followed by 40 cycles of denaturation at 95 °C for 15 s, annealing for 30 s and extension at 72 °C for 30 s. A melt curve was incorporated at the end of each reaction to ensure the specificity of the product. Relative expression analysis was performed using the 2^−∆∆*C*t^ calculation method by normalization to the housekeeping gene *GAPDH*. 

### 4.5. Methylation-Specific PCR (MSP-PCR)

DNA methylation levels of all four members of the *TBX2* subfamily in NSCLC cell lines was analyzed using the Epitect Methyl II DNA restriction kit (QIAGEN, Cat. #335452) according to the manufacturer’s instructions. Reaction mixtures without enzymes were prepared consisting of 250 ng of genomic DNA with 26 µL restriction digestion buffer (5×) and RNase-DNase free water for a final volume of 120 µL. Restriction digestion reactions and enzymes were prepared according to the manufacturer’s instructions. The PCR cycling protocol was composed of an initial denaturation step at 95 °C for 10 min, followed by 3 cycles at 99 °C for 30 s and 72 °C for 1 min, succeeded by 40 cycles of 97 °C for 15 s and 72 °C for 1 min. 

### 4.6. Lung Cancer Cell Treatment with 5-Azacytidine

#### 4.6.1. 5-Azacytidine Preparation

5-azacytidine (Aza) was obtained from Sigma-Aldrich (SIGMA A2385). This agent is water insoluble; as such it was dissolved in DMSO in the dark and aliquoted in amber micro-centrifuge tubes to a final stock concentration of 10 mM and stored at −20 °C. All Aza treatments were carried out in minimal light. Cells were initially seeded in different cell culture plates based on downstream assays and in cell culture medium with 10% FBS. The next day, media was aspirated and cells were incubated in cell culture media with reduced serum (1% FBS). Treatment with Aza was then performed the following day in reduced serum cell culture media (1% FBS) and was replenished every other day as Aza is not stable [14].

#### 4.6.2. MTT Assay

Cellular proliferation of NSCLC cell lines was assessed using the 3-(4,5-dimethylthiazol-2-yl)-2,5-diphenyltetrazolium bromide (MTT) assay. Cells were seeded in 96-well plates and treated with DMSO or varying concentrations of Aza: 1 µM, 5 µM and 10 µM for three time points (24, 48 and 72 h) post-treatment. Cells were incubated with 10 µL MTT dye, followed by the addition of 100 µL stop solution three hours later and then cells were incubated overnight at 37 °C. Absorbance was measured on a Multiskan EX ELISA Reader at 595 nm according to the manufacturer’s instructions. All assays were performed in technical triplicates.

#### 4.6.3. Trypan Blue Exclusion Assay

Cells were seeded in 96-well plates and treated with Aza according to IC50 measurements (1 μM) obtained from MTT assays. Cell numbers were quantified at 24 h and 72 h post-treatment. Cells were diluted in a 1:1 ratio with 0.4% trypan blue solution (Sigma-Aldrich) and counted using a hemocytometer chamber. 

### 4.7. Transfection with Small Interfering RNAs

Transfection of non-treated and Aza-treated H1299 cells with control (scrambled) small interfering RNAs (siRNAs) or siRNAs specific to the *TBX* genes was performed using Lipofectamine RNAiMAX from Invitrogen according to the manufacturer’s instructions. Cells were seeded in six cm plates at a density of 250,000 cells/plate using complete growth medium. Cells were transfected with the siRNAs the following day. The next day, transfected cells were trypsinized and re-seeded onto different plates/dishes to treat with Aza for 24–72 h and then conduct MTT and trypan blue exclusion assays as well as qRT-PCR analysis of the *TBX* genes in the manner described above.

### 4.8. Statistical Analysis

Statistical analyses of differences in *TBX2* subfamily expression and methylation, as well as differences in cell growth and proliferation, were performed using GraphPad Prism 7 software. In order to determine the statistical significance in the difference of means between two experimental data sets, a paired two sample for means Student’s *t*-test was performed. All *p-*values < 0.05 were considered statistically significant.

## 5. Conclusions

In conclusion, we demonstrated that the *TBX2* subfamily of evolutionarily conserved transcription factors, particularly *TBX4* and *TBX5*, are epigenetically suppressed by hypermethylation in human NSCLC. We also showed that *TBX2* subfamily hypermethylation is a common attribute in NSCLC tumors and cell lines. We also found that members of the subfamily, particularly *TBX4* and *TBX5*, are commonly suppressed and hypermethylated in human NSCLC cell lines. Our study also revealed that treatment with the anti-tumor drug and DNA methyltransferase inhibitor 5-azacytidine markedly increased expression levels of all four members of the subfamily concomitantly with reduced NSCLC cell growth and proliferation. Lastly, we preliminarily showed that collective induced expression of the four members of the *TBX2* subfamily is important for the anti-growth effects of Aza in NSCLC cells. Our findings provide new insights into the molecular (epigenetic) pathogenesis of NSCLC and suggest that *TBX2* subfamily methylation may serve as a potential biomarker for early detection and intervention of this morbid disease.

## Supplementary Materials

Supplementary materials can be found at https://www.mdpi.com/1422-0067/20/5/1159/s1.

## Abbreviations

Aza	5-azacytidine
DMEM	Dulbecco’s modified eagle media
DMSO	dimethyl sulfoxide
FBS	fetal bovine serum
GAPDH	glyceraldehyde 3-phosphate dehydrogenase
LUAD	lung adenocarcinoma
LUSC	lung squamous cell carcinoma
MSP	methylation specific PCR
NSCLC	non-small cell lung cancer
PBS	phosphate buffered saline
siRNA	small interfering RNA
TBX	T-box gene
TCGA	The Cancer Genome Atlas
