# Supplementary material for: Epigenetic Suppression of the T-box Subfamily 2 (TBX2) in Human Non-Small Cell Lung Cancer
Source: Int J Mol Sci. 2019 Mar 7;20(5):1159. doi: 10.3390/ijms20051159 (PMC6429281; doi:10.3390/ijms20051159)
Supplement: Supplementary file 1 [file ijms-20-01159-s001.pdf]

Epigenetic suppression of the T-box subfamily 2 (*TBX2*) in human non-small cell lung cancer

Eliana Nehme, Zahraa Rahal, Ansam Sinjab, Athar Khalil, Hassan Chami, Georges Nemer, Humam Kadara

SUPPLEMENTARY TABLES

Table S1: Members of the *TBX2* subfamily are hyper-methylated in NSCLC cell lines.

**Genes**	***TBX4***		***TBX5***	
	**UM (%)**	**M (%)**	**UM (%)**	**M (%)**
**H1299**	0	100	0.37	99.63
**H1693**	0	100	48.09	51.91
**H1792**	0.31	99.69	31.64	68.36
**H23**	0.04	99.96	0	100
**H3255**	0.21	99.79	99.95	0.05
**H460**	0.04	99.96	0.75	99.25
**H1650**	0.33	99.67	0.14	99.86
**HCC827**	0	100	26.42	73.58
**NAC**	98.73	1.27	98.8	1.2

UM, unmethylated fraction; M, methylated fraction; NAC, normal alveolar cells

Table S2: Primer sequences for *TBX2*, *TBX3*, *TBX4*, *TBX5* and *GAPDH* PCR analysis.

Gene	Sequence (5'-3')	Reference
TBX2	F-AGTGGATGGCTAAGCCTG	(Z. Zhang & Guo, 2014)
	R- ACGGGTTGTTGTCGATC	
TBX3	F-GAGGCTAAAGAACTTTGGGATCA	Primer bank
	R- CATTTCGGGGTCGGCCTTA	
TBX4	F- CACTACCAGCACGAGAAC	(Horie et al., 2017)
	R- CCAGATAGGATCGCTTGC	
TBX5	F- AAGTAAAGAATATCCCGTGGTC	(R. Ma et al., 2017)
	R- AGACTCGCTGCTGAAAGG	
GAPDH	F- GTCAGTGGTGGACCTGACCT	(Pasini et al., 2007)
	R- TCGCTGTTGAAGTCAGAGGA	

References

1. Zhang, Z., & Guo, Y. (2014). High TBX2 expression predicts poor prognosis in non-small cell lung cancer. *Neoplasma*. doi:10.4149/neo_2014_059
2. Horie, M., Miyashita, N., Mikami, Y., Noguchi, S., Yamauchi, Y., Suzukawa, M., . . . Nagase, T. (2017). TBX4 is involved in the super-enhancer-driven transcriptional programs underlying features specific to lung fibroblasts. *American Journal of Physiology-Lung Cellular and Molecular Physiology, 314*(1), L177-L191. doi:10.1152/ajplung.00193.2017
3. Ma, R., Yang, Y., Tu, Q., & Hu, K. (2017). Overexpression of T-box Transcription Factor 5 (TBX5) Inhibits Proliferation and Invasion in Non-Small Cell Lung Carcinoma Cells. *Oncology research, 25*(9), 1495-1504. doi:10.3727/096504017x14883287513729
4. Pasini, B., McWhinney, S. R., Bei, T., Matyakhina, L., Stergiopoulos, S., Muchow, M., . . . Stratakis, C. A. (2007). Clinical and molecular genetics of patients with the Carney–Stratakis syndrome and germline mutations of the genes coding for the succinate dehydrogenase subunits SDHB, SDHC, and SDHD. *European Journal Of Human Genetics, 16*, 79. doi:10.1038/sj.ejhg.5201904.
